# Comparative Effects of ZnO, MgO, and CaO Nanoparticles in 3D‐Printed Chitosan–Agarose Scaffolds on Antibacterial and Osteogenic Outcomes

**DOI:** 10.1002/mabi.202500232

**Published:** 2025-09-22

**Authors:** Amir Hashemi, Masoumeh Ezati, Rima Paul, Inna Zumberg, Jaromir Bacovsky, Zdenka Fohlerova, Valentyna Provaznik

**Affiliations:** ^1^ Department of Biomedical Engineering Faculty of Electrical Engineering and Communication Technologies Brno University of Technology Brno Czech Republic; ^2^ Delong Instruments a.s. Brno Czech Republic; ^3^ Department of Microelectronics Faculty of Electrical Engineering and Communication Brno University of Technology Brno Czech Republic

**Keywords:** antibacterial properties, bioprinted scaffolds, bone marrow stem cells, Chitosan‐agarose biomaterial ink, extrusion 3D bioprinting, osteogenic differentiation, ZnO/MgO/CaO nanoparticles

## Abstract

In the field of orthopedic surgery, large bone defects resulting from trauma, surgical resection, or congenital anomalies present significant challenges. In many cases, treatment necessitates scaffold structures that not only support bone regeneration but also address potential bacterial infections that can impede healing. In this study, we developed 3D bioprinted scaffolds using hydrogel‐based biomaterial ink comprising a blend of chitosan (CS) and agarose (AG), each separately fortified with ZnO, MgO, and CaO nanoparticles (NPs). We performed a comprehensive assessment of the inks' printability and wettability, and ascertained their rheological properties. The in vitro degradation of 3D bioprinted scaffolds was analyzed, their antibacterial capabilities against *Escherichia coli* (*E. coli*) and *Staphylococcus aureus* (*S. aureus*) were explored, and the differentiation of bone marrow mesenchymal stem cells (BMSCs) was evaluated. The findings indicated that the hydrogel, CS‐AG (CA), composed of 3.5% (w/v) CS and 1.5% (w/v) AG, demonstrated superior printing characteristics. Among the nanoparticles, ZnO proved to be a notable booster of antibacterial activity and facilitated osteogenic differentiation and proliferation of bone marrow stem cells. Conversely, MgO showed similar antibacterial efficacy but was less successful in promoting cell proliferation compared to ZnO and CaO, whereas CaO displayed the weakest antibacterial efficacy. The results identify the ZnO NP‐loaded CA biomaterial ink as a viable option for addressing bone abnormalities, enhancing bone repair, and preventing bacterial infection.

## Graphical abstract

3D‐bioprinted chitosan–agarose (CA) scaffolds loaded with calcium oxide (CAC), zinc oxide (CAZ), or magnesium oxide (CAM) nanoparticles (NPs) were developed. The NPs improved antibacterial activity, rheological properties, and bone marrow cell (BMC) proliferation. These composite scaffolds show promise for bone regeneration by enhancing osteogenic differentiation and structural stability.

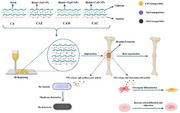

## 1 Introduction

Large bone defects caused by trauma, surgical resection, or congenital anomalies continue to pose a significant challenge in the field of orthopedics. Although bone possesses innate regenerative capacity, extensive defects often exceed the natural ability of the bone to heal without intervention. Conventional treatments, such as autografts, allografts, and xenografts, are limited by morbidity at the donor site, limited availability, and the possibility of disease transmission [1]. Consequently, the development of scaffolds for bone regeneration has attracted considerable interest. In an ideal situation, a scaffold would support osteoconduction, osteoinduction, and osteogenesis and resist potential bacterial infections that can compromise healing [2].

Antibacterial properties within a scaffold are essential, as bacterial colonization and the subsequent formation of biofilm can inhibit bone regeneration and result in implant‐related infections [3]. In the past, antibacterial coatings were applied directly to the implants to prevent bacterial adhesion. However, such coatings often suffer from limited drug release duration, potential toxicity, and diminished long‐term effectiveness due to bacterial resistance [4]. By contrast, 3D scaffold structures can provide a controlled and sustained release of antibacterial agents, ensuring prolonged protection against bacterial colonization. The porous nature of scaffolds also offers more surface area for antibacterial agents to facilitate nutrient and oxygen transport, enhancing efficacy. Furthermore, scaffolds can be designed to degrade in sync with bone formation, providing temporary support that matches the body's natural healing rate. Infusing scaffolds with multiple therapeutic agents enables a synergistic approach to bone regeneration and antibacterial action. This capability makes scaffolds a more versatile and promising alternative to conventional coatings in the field of bone regeneration [5, 6, 7]. Despite the precise control over geometry and porosity afforded by 3D printing, this technique can introduce structural anisotropy, weak interlayer bonding, and other defects that compromise mechanical properties. Adequate mechanical strength is crucial for scaffolds to withstand physiological stresses and maintain structural integrity throughout the healing process [8]. Therefore, evaluating and optimizing the mechanical performance of 3D‐printed scaffolds is essential to meet the biomechanical requirements necessary for effective bone regeneration. This study investigates 3D‐printed CS–AG hydrogel scaffolds enriched with ZnO, MgO, or CaO NPs to address these challenges.

While the antibacterial efficacy of certain NPs (e.g., silver) is well‐established, ZnO, MgO, and CaO are gaining attention in bone regeneration. For example, Christy et al. [9] showed that adding nano‐ZnO to a CS/PVA/nano–bioactive glass composite increased swelling, improved antibacterial effectiveness against E. faecalis, and extended biodegradation time. In another study, adding bioglass and nano‐ZnO to electrospun PLLA scaffolds slowed hydroxyapatite formation and endowed scaffolds with antibacterial properties (reducing S. aureus viability by 60% in 6 h) [10]. Pan et al. [11] used a Pickering emulsion to create macroporous GelMA–PEG hydrogels with MgO NPs; the macroporosity enhanced nutrient transport and cell communication, and Mg^2+^ release promoted osteogenic differentiation of BMSCs, resulting in improved bone regeneration in vivo. Likewise, incorporating CaO NPs into electrospun PCL/gelatin matrices improved osteogenic differentiation (elevated ALP expression) and cell viability, though antibacterial outcomes were inconsistent [12].

Recent studies have begun to embed metal oxide NPs into 3D‐printed scaffolds to enhance functionality. Safiaghdam et al. [13] developed 3D‐printed PCL/β‐TCP scaffolds loaded with MgO NPs, which significantly improved mechanical properties and osteogenic potential, boosting cell proliferation in vitro and bone formation in vivo. Similarly, 3D‐printed ADA–GEL hydrogels with silica–calcia NPs were designed for sustained release of an osteogenic drug (icariin), enhancing bone regeneration [14]. Other research combining ZnO or various metal oxide NPs in 3D‐printed scaffolds has shown enhanced mechanical properties, bioactivity, and antibacterial efficacy, leading to improved bone healing [15]. However, a direct head‐to‐head comparison of ZnO, MgO, and CaO within the same 3D‐bioprinted scaffold system has been lacking.

Due to its inexpensive price, low immunogenicity, biodegradability, biocompatibility, and especially its antibacterial characteristics, CS is widely employed in tissue regeneration applications. Numerous studies have demonstrated its efficacy for wound healing and osseointegration. However, CS has a limited mechanical strength and degrades quickly [16]. AG, a naturally occurring polysaccharide, offers potential as a bio‐ink for 3D printing due to the simplicity with which its mechanical properties may be tuned, its capacity for chemical functionalization, and its quick gelation kinetics. However, AG hydrogels lack the rheological characteristics required for bioprinting applications and have limited bioactivity [17]. Table 1 provides a comparative overview of key studies using oxide NPs in bone tissue engineering, underscoring the novelty of our work (combining chitosan–agarose hydrogel, multiple NP types, extrusion bioprinting, and a broad evaluation of printability, degradation, antibacterial, and osteogenic outcomes). Whereas earlier studies demonstrated individual benefits of ZnO, MgO, or CaO in varied systems, this work is the first to compare all three within a single 3D‐printed CS–AG scaffold with comprehensive characterization. Our study aims to fill this gap by systematically evaluating ZnO vs. MgO vs. CaO in the same scaffold platform.

**TABLE 1:** Comparison of representative studies using metal oxide NPs in bone tissue engineering scaffolds.

Study	Base Material(s) and NP(s)	Fabrication method	Key characterizations/Outcomes	Main findings	Gap left/how our study is novel
**Christy et al**. [9]	Chitosan–PVA + ZnO	Freeze‑drying	Swelling, degradation, antibacterial	ZnO ↑ swelling & antibacterial efficacy	Not 3‑D printed; single NP; no rheology, printability or osteogenic data
**Pan et al**. [11]	GelMA–PEG + MgO	Macroporous emulsion hydrogel (no printing)	Macropore imaging; ALP; in vivo bone	MgO ↑ BMSC osteogenesis	No antibacterial data; not 3‑D printed; different polymer system
**Münchow et al**. [12]	PCL / Gelatin + CaO	Electrospinning	Fiber size; antibacterial; ALP	CaO ↑ osteogenesis; mixed antibacterial	Fibrous scaffold; not hydrogel; no printing; single NP
**Safiaghdam et al**. [13]	PCL + β‑TCP + MgO	Extrusion 3‑D printing (rigid)	Mechanical; in vivo bone	MgO ↑ stiffness and bone formation	Rigid polymer–ceramic; no hydrogel, swelling, or antibacterial data; single NP
**Monavari et al**. [14]	Alginate‑dialdehyde / Gelatin + SiO_2_‑CaO	Extrusion 3‑D printing hydrogel	Drug release; cell viability	Ca‑Si NPs ↑ drug‑loaded osteogenesis	No ZnO or MgO; no antibacterial assay; no NP comparison
**Bose et al**. [15]	3‑DP tricalcium‑phosphate + SiO_2_ / ZnO	Binder‑jet 3‑D printing (ceramic)	Angiogenesis; osteogenesis	ZnO ↑ bone & vessel formation	Ceramic scaffold; not hydrogel; no antibacterial test; single NP
^**^This work^**^	Chitosan‑Agarose + ZnO/MgO/CaO (0.5 % w/v)	Extrusion 3‑D bioprinting (macro‑porous lattice)	Rheology and printability; swelling; mechanical; 4‑week degradation; antibacterial; BMSC proliferation; ALP; qPCR; mineralization; SEM (degradation and infiltration)	Balanced printability; all NPs ↑ stiffness and ↓ swelling; **ZnO best antibacterial and osteogenic**; stable structure;	First head‑to‑head comparison of ZnO, MgO, and CaO in the same 3‑D‑printed chitosan‑agarose hydrogel with full printability, degradation, antibacterial, and osteogenic data.**

This work utilized 3D‐printed CS–AG scaffolds (optimized ink: 1.5% AG + 3.5% CS) to comparatively assess how 0.5% (w/v) of ZnO, MgO, or CaO NPs influence antibacterial properties, cell interactions, and bone regeneration. We evaluated ink printability, rheological properties, in vitro degradability, and surface wettability. Our results identify ZnO‐NP–doped scaffolds as particularly promising for enhancing bone regeneration while reducing bacterial infections during bone healing.

## 2 Materials and Methods

### 2.1 Preparation of Biomaterial Ink

The base hydrogel ink, referred to as CA, consisted of chitosan (medium molecular weight) and agarose with concentrations of 3.5% and 1.5% (w/v), respectively. Both chitosan and agarose were obtained from Sigma–Aldrich. We used chitosan of medium molecular weight (∼200 kDa, 75–85% degree of deacetylation) and agarose (low electroendoosmotic agarose, average molecular weight ∼120 kDa). To prepare the CA hydrogel, 0.35 g of chitosan (CS) was dissolved in 10 mL of distilled water with 1% (v/v) acetic acid, stirring at 500 rpm for 4 h to ensure complete dissolution of chitosan. Then, 0.15 g of agarose (AG) was gradually added to the chitosan solution. The mixture was incubated in a 50°C water bath with continuous stirring (200 rpm) for 24 h, yielding a uniform CS‐AG hydrogel. After this base hydrogel was formed, the oxide nanoparticles were incorporated: we added ZnO, MgO, or CaO nanopowders (synthesized in‐house, see below) at the predetermined concentration (0.5% w/v, i.e., 50 mg NP per 10 mL hydrogel). The NPs were added after the chitosan‐agarose blend was homogeneous but still warm (∼50°C), and the mixture was stirred vigorously for 30 min. We additionally bath‐sonicated the NP–hydrogel mixture for 2 min to promote uniform dispersion of the NPs and to break up any agglomerates. This process produced the nanoparticle‐fortified inks: CAZ (with ZnO), CAM (with MgO), and CAC (with CaO). All inks were loaded into 3D printer cartridges at ∼50°C for subsequent printing. Table 2 (formulation details) lists the composition of each hydrogel ink. We selected 0.5% w/v as the nanoparticle concentration for final scaffolds based on preliminary tests of printability and scaffold integrity. This “medium” concentration provided a good balance of structural support and bioactivity without clogging the printer nozzle or causing structural collapse. Lower (0.3%) and higher (0.7%) NP loadings were initially screened; 0.5% was found to be optimal, and all results reported are for that concentration in each case.

**TABLE 2:** Formulation of CA hydrogel inks augmented with NPs for enhanced 3D printing. Concentrations of each NP (0.5% w/v) were optimized for scaffold integrity and printability.

No.	Sample	w/v (CS)	w/v (AG)	w/v (acetic acid)	w/v (NPs)
**1**	CA	3.5%	1.5%	1%	0
**2**	CAZ	3.5%	1.5%	1%	0.5%
**3**	CAC	3.5%	1.5%	1%	0.5%
**4**	CAM	3.5%	1.5%	1%	0.5%

### 2.2 Printability

Scaffold designs were created in Autodesk Fusion 360 and saved as STL files. G‐code toolpaths were generated using CELLINK HeartWare software and loaded into an INKREDIBLE+ extrusion 3D bioprinter (CELLINK, Gothenburg, Sweden). We designated each ink formulation with a unique code for clarity: CA (no NPs), CAZ (ZnO NPs), CAC (CaO NPs), and CAM (MgO NPs). These designations are used consistently in reporting results. Printing parameters (extruder temperature, pressure, speed, needle gauge) were carefully calibrated for each ink to ensure consistent filament extrusion (see Table 3 for parameters). Table 4 presents images of representative printed scaffolds (3D lattice infill pattern) produced with CA and NP‐doped inks.

**TABLE 3:** Printing parameters for the fabrication of pure CA scaffold and NPs (0.5% w/v) doped CA scaffolds.

No.	Sample	Extruder temperature (±1°C)	Printing pressure (MPa)	Printing speed (mm/s)	Needle diameter (mm)
**1**	CA	25	0.10	5	0.41
**2**	CAZ	40	0.15	3	0.41
**3**	CAC	40	0.15	3	0.41
**4**	CAM	40	0.20	3	0.41

**TABLE 4:** Digital image of different 3D printed scaffolds and the desired infill pattern obtained by the software.

The desired 3D structure infill pattern	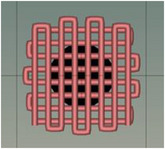			
Different scaffold samples	CA	CAM	CAC	CAZ
**Images of 3D printed scaffolds**	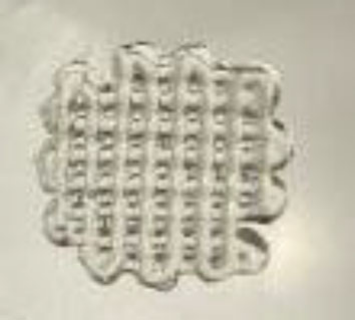	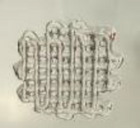	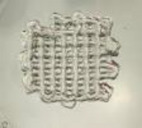	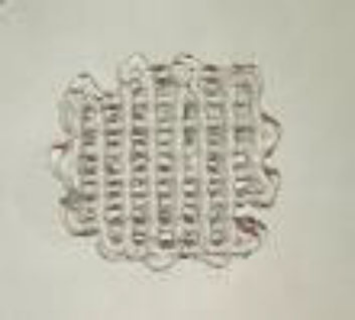

### 2.3 Synthesis of PVP‐capped NPs

#### 2.3.1 ZnO NPs

Synthesis of ZnO NPs was carried out following a wet chemical technique. We prepared 0.80 m potassium hydroxide (KOH) solution in 60 mL distilled water and ultrasonicated (Elmasonic P 30H) it at 50°C for 30 min. On the other hand, a 0.20 m solution of zinc nitrate was prepared by dissolving zinc nitrate hexahydrate [Zn (NO_3_)_2_·6H2O] salt in 40 mL of distilled water. Under constant sonication, KOH solution was added dropwise to the zinc nitrate solution until we attained pH 8. Polyvinylpyrrolidone (PVP) was used as a capping agent to restrict the agglomeration of the NPs. The white precipitate formed was collected using a centrifuge (Hettich MIKRO 200) and washed thoroughly using distilled water. The synthesized powder sample was then dried under an infrared (IR) lamp for an hour, followed by drying in a Muffle furnace (Thermo Scientific, Thermolyne) at a temperature of 450°C for 1 h.

#### 2.3.2 MgO NPs

A 0.20 m of magnesium nitrate hexahydrate solution [Mg (NO_3_)_2_ 6H_2_O] was prepared in 160 mL of distilled water. KOH solution (0.80 m) containing PVP was added dropwise to the magnesium nitrate solution and sonicated at 50°C till the formation of a white precipitate of magnesium hydroxide. The white gel‐like precipitate was collected through centrifugation, washed thoroughly using distilled water, and allowed to dry under an IR lamp. The dried white powder was then calcined at 900°C for 2 h in the Muffle to get MgO NPs.

#### 2.3.3 CaO NPs

CaO NPs were synthesized following a similar wet chemical technique as in the case of MgO NPs. A 0.20 m of calcium nitrate tetrahydrate solution [Ca (NO_3_)_2_ 4H_2_O] was prepared in 160 mL of distilled water. A 0.80 m KOH solution containing PVP was added dropwise to the calcium nitrate solution and sonicated at 50°C till the formation of a white precipitate. The precipitate of Ca (OH)_2_ formed was centrifuged and rinsed several times with distilled water. After drying under the IR lamp, the white powder was heat‐treated at 900°C for 2 h in the Muffle furnace to obtain CaO NPs. All chemicals used for the synthesis of nanoparticles were from MERCK (Reagent grade) and were used without further purification.

### 2.4 Characterization of NPs and Biomaterial Ink

The morphological analysis of the synthesized nanoparticles was performed using a Tescan Mira II scanning electron microscope (SEM) and a low‐voltage electron microscope, LVEM 25E from Delong Instruments, Czech Republic, working at an energy of 25 keV. The chemical compositions of NPs were confirmed from the energy dispersive X‐ray analysis using an EDS detector attached to the Tescan Mira II. The crystal structures of the NPs were determined with the help of an X‐ray powder diffractometer (Rigaku Smartlab 3 kW).

The rheological properties of biomaterial ink formulations were assessed using a Discovery Hybrid Rheometer‐2 (TA Instruments, USA), equipped with a 40 mm parallel plate geometry set at a 500 µm gap. The trials were carried out at a regulated temperature of 25 and 40°C, for the CA and the NPs doped CA samples, respectively, to simulate the thermal conditions encountered during the ink extrusion process. Frequency sweeps were conducted at a 1% deformation level, spanning an angular frequency range of 0.628 to 125.704 rad/s to determine the values of storage modulus, loss modulus, and complex viscosity. The chemical compositions of the biomaterial inks were examined through Fourier Transform Infrared Spectroscopy (FTIR) and X‐ray Photoelectron Spectroscopy (XPS). The FTIR spectra were obtained using a Vertex 70v vacuum spectrometer (Bruker, Germany) equipped with an Attenuated Total Reflectance (ATR) module spectroscopy in the range of 500–4000 cm^−1^. XPS was performed to analyze the surface elements using an Axis Supra spectrometer (Kratos Analytical Ltd., UK). The water contact angle (WCA) was determined using the sessile droplet method with a See System (Advex Instruments) on a thin film of each biomaterial ink placed on a glass Petri plate. The contact angle determination technique involved dropping small droplets (3 µL) of deionized water onto the surface and measuring the angle between the droplet and the surface for 3 s.

### 2.5 Hydrolytic Degradability and Swelling

A gravimetric measurement was performed to evaluate the in vitro degradation rate of each scaffold for a period of 4 weeks. The samples were incubated in 4 mL of PBS solution at a pH of 7.4 and a temperature of 37°C. At weekly intervals, the samples were removed from the PBS solution, rinsed with distilled water, and dried in a vacuum oven at 25°C. The changes in the weight of the sample were then calculated using Equation (1)

(1) $$\mathrm{Degradation}\;\left(\%\right)=\frac{W_{0-}W_{t}}{W_{0}}\times 100$$

where, *W_0_* is the sample's weight before soaking into PBS, and *W_t_* is the sample's weight after a specific soaking time.

To determine the swelling ratio, small fragments from each scaffold group were immersed in PBS (pH 7.4) at 25°C until they reached equilibrium weight. The initial dry weight (Wi) of each sample was recorded prior to immersion. At predetermined time intervals, the samples were removed, gently blotted with filter paper to remove surface moisture, and weighed to obtain the wet weight (Ww). The swelling percentage was calculated using the following Equation (2)

(2) $$\mathrm{Swelling}\;\left(\%\right)=\frac{W_{w-}W_{i}}{W_{i}}\times 100$$

All measurements were performed in triplicate, and the mean values were reported.

The cross‐linking density (Ve, mol/cm^3^) of the 3D‐printed chitosan–agarose hydrogels was calculated based on the Flory–Rehner theory, which relates the equilibrium swelling behavior of a hydrogel to its network structure. Following the method described by [18], the calculation involved the following steps: After lyophilization, each scaffold sample was weighed (dry weight, Wd), then fully immersed in deionized water at room temperature for 48 h to reach equilibrium swelling. The swollen weight (Ws) was then recorded. The equilibrium swelling ratio (Q) was calculated using Equation (3):

(3) $$Q=\frac{W_{s}}{W_{d}}$$

Next, the polymer volume fraction in the swollen state (Vr) was determined according to Equation (4):

(4) $$V_{r}=\frac{1}{1+Q}\;$$

Finally, the cross‐linking density (Ve) was calculated using the Flory–Rehner Equation (5):

(5) $$V_{e}=\frac{-ln\left(1-V_{r}\right)+V_{r}+\chi_{1}V_{r}^{2}}{Vs\;\times \;Vr^{1/3}}\;$$

where: χ is the polymer–solvent interaction parameter, taken as 0.55, representing an average for hydrophilic systems like chitosan and agarose in water [19, 20]. Vs is the molar volume of water (18 cm^3^/mol). This method allowed estimation of the cross‐linking density of each hydrogel formulation based on its swelling behavior.

### 2.6 Antibacterial Properties

The antibacterial properties of the scaffolds were evaluated using an adapted in vitro quantitative methodology against *E. coli* (ATCC 25922) and *S. aureus* (ATCC 25923). The scaffold samples were subjected to bacterial suspensions in Luria‐Bertani (LB) broth at a concentration of 1 × 10^6^ colony‐forming units per mL (CFU/mL). The control (CA scaffold without NPs) and bacterial suspension with scaffolds were placed into the incubator at 37°C and cultured overnight under a gentle stirring at a speed of 120 rpm. Then, the solutions were diluted (1:10^8^) in PBS and spread onto LB agar to promote the growth of colonies at a temperature of 37°C for 24 h. The quantification of colonies, denoting CFUs, was conducted by counting the colonies formed on the agar plates. The antibacterial efficacy was determined by comparing the number of colonies in the control group to the number in the presence of the scaffolds.

The antibacterial activity of the scaffolds against *E. coli* and *S. aureus* was further evaluated by the agar disc diffusion method. A bacterial suspension with a concentration of 1.5 × 10^8^ CFU/mL was evenly spread over the surface of Mueller‐Hinton agar (MHA) plates. Sterile samples with 13 mm diameter and a control were placed on the surface of the inoculated MHA. The plates were incubated at 37°C for 24 h. The measurement of the diameter of inhibition zones surrounding the samples and the antibiotic disc was conducted using ImageJ software. The distance from the edge of each scaffold sample and antibiotic disc to the end of the clear zone was measured in 12 different directions, with 3 measurements taken on each side of the scaffold sample. The mean inhibition zone length, which serves as a quantifiable measure of antibacterial activity, was determined by averaging these 12 measurements.

### 2.7 In Vitro Cellular Studies

#### 2.7.1 Cell Culture

Bone marrow mesenchymal stem cells (BMSCs; passage 3) were obtained from Sigma‐Aldrich (Germany). The cells were cultured in DMEM supplemented with 10% fetal bovine serum (FBS) and 1% penicillin/streptomycin at 37°C in a 5% CO_2_ atmosphere. The culture medium was replaced every 2 days. The cells were harvested for the experiments using trypsinization and transferred to a fresh culture medium.

#### 2.7.2 Cell Proliferation

MTT (3‐(4,5‐dimethylthiazol‐2‐yl)‐2,5‐diphenyl tetrazolium bromide) assay was performed to examine cell proliferation on the printed scaffolds. The samples were sterilized with a UV light for 20 min and placed in a 24‐well plate. The scaffolds were seeded with 15,000 cells/mL incubated for 1, 3, and 5 days at 37°C. After the designated time points, the scaffolds were rinsed with PBS to remove any unadhered cells and placed on a new well plate. A solution of 5 mg/mL MTT was added to each sample and incubated for 3 h. Then, the MTT solution was replaced with DMSO to dissolve the insoluble formazan crystals. The optical density was measured using a plate reader at 570 nm.

#### 2.7.3 Cell Morphology

The morphology of BMSCs cultured on the samples was analyzed using a Leica TCS SP8 X confocal microscope (Leica Microsystems, Wetzlar, Germany). The sterilized samples were placed in confocal dishes, seeded with 5000 cells, and incubated for 2 days. After the incubation period, the samples were rinsed three times with PBS. CellTracker Green CMFDA (5‐chloromethylfluorescein diacetate) from Thermo Fisher Scientific (Waltham, MA, USA) was used to monitor the morphology of the cells by staining the cell cytoplasm. The final staining solution, prewarmed to 37°C (5 µm of CMFDA), was added to the samples and incubated for 15 min. The samples were rinsed one more time with PBS, and fresh medium was added. The stained cells were then observed under the confocal microscope.

#### 2.7.4 Osteogenic Differentiation Assessment

To examine the process of osteoblastic differentiation, the activity of alkaline phosphatase (ALP), mineralization, and the expression of osteocalcin were assessed. The ALP activity on the scaffold samples was evaluated for up to 21 days using a colorimetric ALP test kit (Abcam plc, Cambridge, UK). Briefly, the scaffolds were washed three times with PBS, and BMSCs were removed from the surface by trypsinization. The cell suspension was centrifuged, and the cell pellet was washed with cold PBS. A 160 µL ALP buffer was added to the pellet and sonicated on ice for 5 min. The cell lysates were further centrifuged at 4°C for 15 min at 15,000 rpm to remove any cell debris. Subsequently, 80 µL of the cleared lysate was mixed with 50 µL of 5 mm p‐nitrophenyl phosphate (pNPP) in a 96‐well plate. The mixture was left to incubate at room temperature for 1 h, and then, the enzymatic process was terminated by the addition of 20 µL of stop solution. The rate of p‐nitrophenol formation, measured photometrically at 405 nm, is proportional to the catalytic concentration of ALP present in the sample. ALP activity (U/L) in the samples was calculated as recommended by the manufacturer.

Quantification of osteocalcin gene expression in BMSCs seeded on the scaffolds was evaluated using qPCR on day 21. RNA was isolated using a RNeasy Mini Kit (Qiagen, Hilden, Germany). The concentration of the extracted RNA was measured via NanoPhotometer (IMPLEN, Munich, Germany) before generating a RevertAid First Strand cDNA Synthesis Kit (Thermo Fisher, Waltham, Massachusetts, US). Quantification of osteocalcin (Forward: GGCGCTACCTGTATCAATGG, Reverse: TCAGCCAACTCGTCACAGTC) was performed using a LightCycler 480 High Resolution Melting Master SYBR green amplification kit (Roche, Basel, Switzerland) in croBEE RT‐PCR System (GeneProof, Brno, Czech Republic). The PCR conditions were set at 95°C for 5 min, followed by 35 cycles of amplification at 95°C for 30 s, 60°C for 30 s, and 72°C for 30 s. GAPDH (Forward: GGTCGGAGTCAACGGATTTG, Reverse: ATGAGCCCCAGCCTTCTCCAT) was used as the housekeeping gene for all trials. The relative quantification of osteocalcin expression was determined using the ΔΔCt method. A total of n = 3 samples were analyzed from each treatment group.

To Evaluate the Presence of Calcium Deposits on the Scaffolds, Alizarin Red S (ARS) Staining Was Conducted, Following a Methodology Previously Described [21]. Briefly, the Scaffolds Seeded With 25,000 Cells Were Rinsed Twice With PBS After the 14^th^ Day Culture Period. Then, the Cells Were Fixed With 4% Paraformaldehyde for 20 Min at Room Temperature. Subsequently, They Were Then Washed With Deionized Water and Treated With a 40 mm ARS Solution (Sigma‐Aldrich, St. Louis, MS, USA) in the Dark for 30 Min. After Incubation, the Samples Were Rinsed With Distilled Water to remove any Residual Alizarin Dye. The Degree of Mineralization Was Captured by Imaging the Scaffolds Using a Phase Contrast Microscope (Nikon, Eclipse E200, Tokyo, Japan)

### 2.8 Statistical Analysis

The experiments were conducted in triplicate, and results are presented as mean ± standard error of the mean. Group comparisons were performed using one‐way analysis of variance (ANOVA), with statistical significance set at ^*^ *p* < 0.05.

## 3 Results and Discussion

### 3.1 Morphology and Composition of the NPs

**FIGURE 1:**
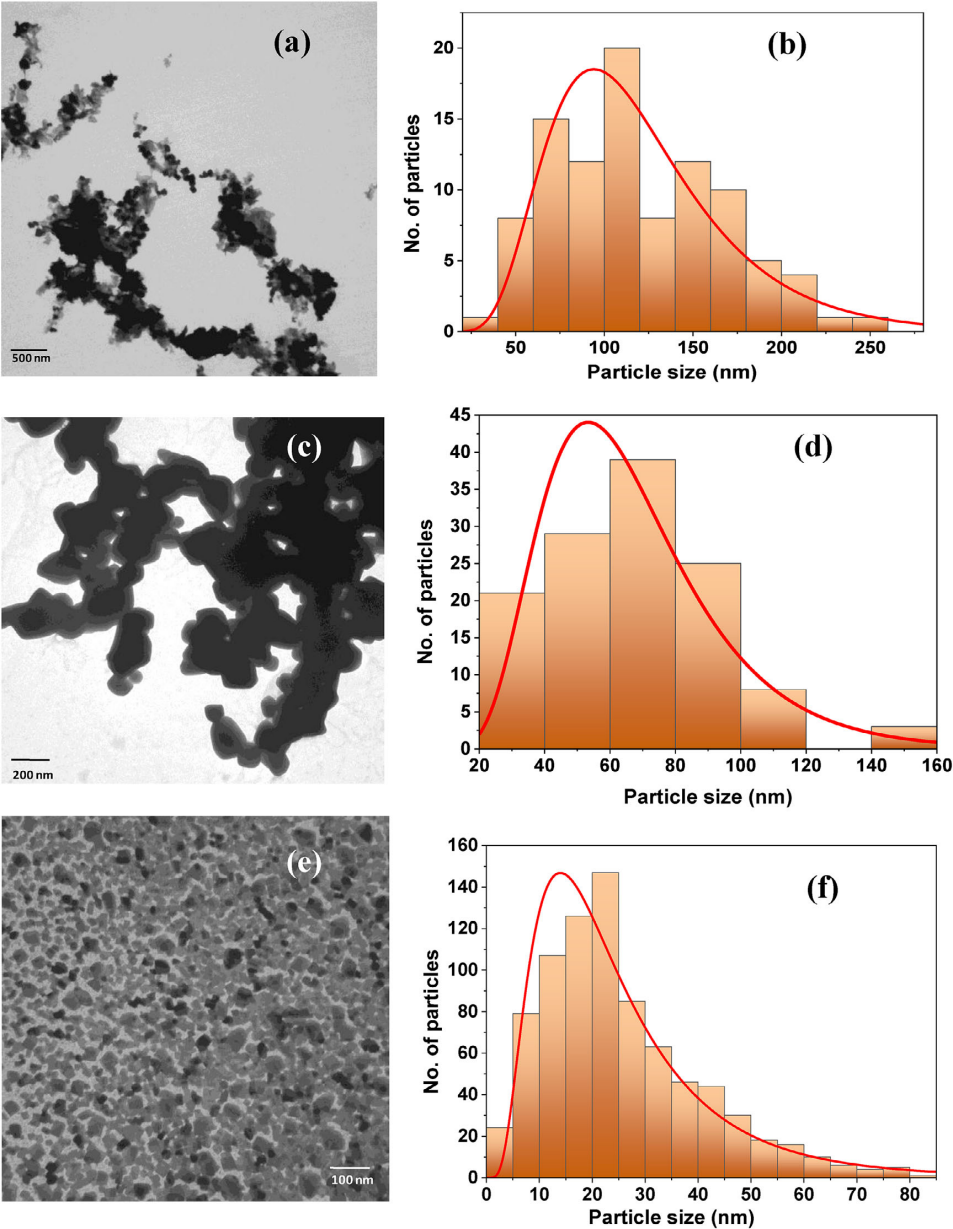
TEM micrographs of (a) ZnO, (c) MgO, and (e) CaO nanoparticles. Corresponding particle size distributions with lognormal fits are shown in (b), (d), and (f), respectively.

**FIGURE 2:**
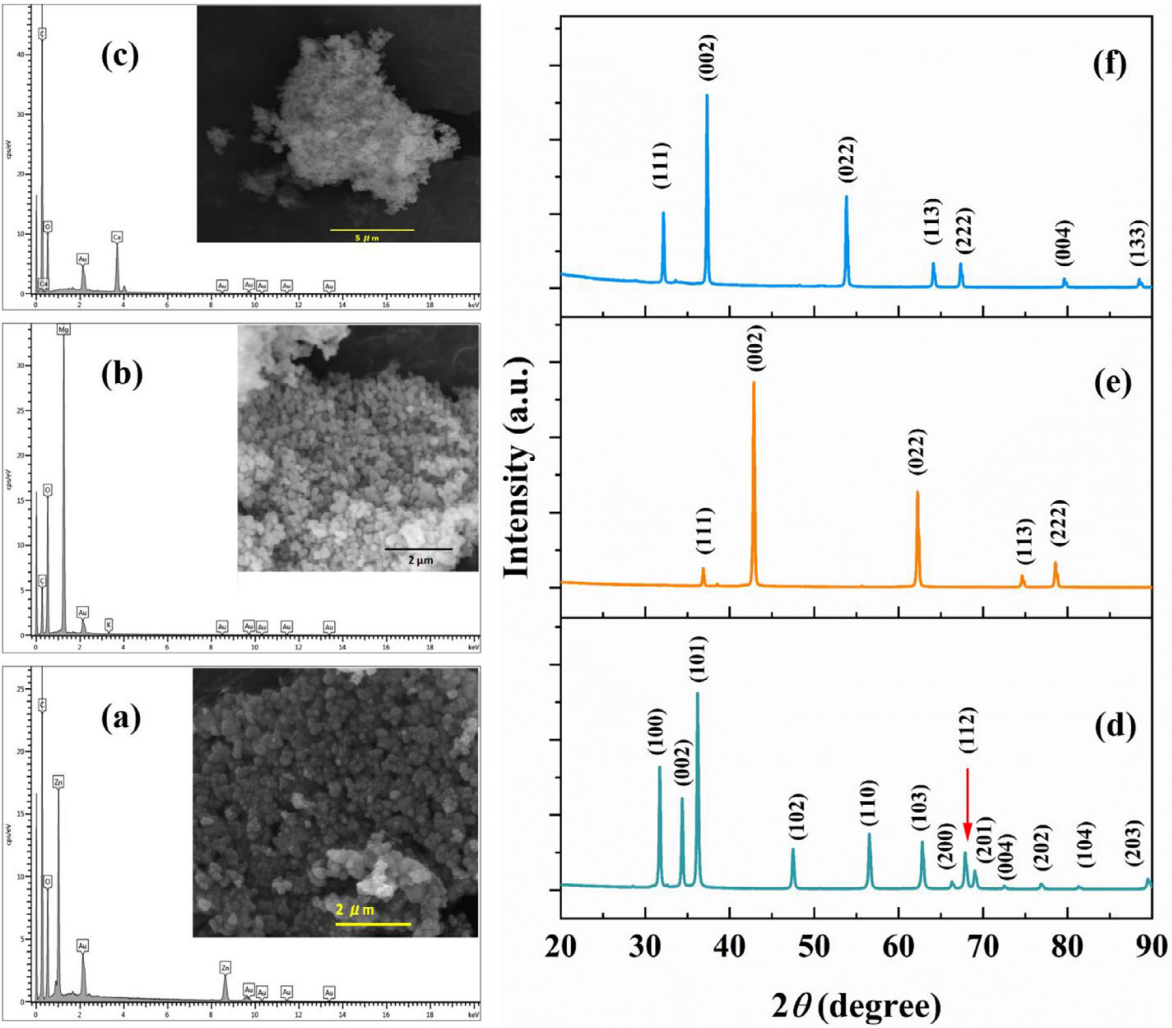
EDS spectra of (a) ZnO, (b) MgO, and (c) CaO nanoparticles with their corresponding SEM micrograph as insets; XRD patterns of the synthesized (d) ZnO, (e) MgO, and (f) CaO NPs are displayed.

The bright field TEM micrographs of the synthesized nanostructured ZnO, MgO, and CaO particles are shown in Figure 1a,c, and 1e respectively. Particle sizes were measured using ImageJ software, and the corresponding size histograms are presented in Figures 1b for ZnO, 1(d) for MgO, and 1(f) for CaO. The average particle sizes for ZnO, MgO, and CaO obtained with lognormal curve fitting are approximately ∼ 53, 93, and 14 nm, respectively. The EDS spectra of the NPs confirm their compositions as shown in Figures 2a for ZnO, 2(b) for MgO, and 2(c) for CaO, with their respective SEM micrograph in the insets. The presence of C is attributed to the PVP capping of the NPs. Au arises from the gold coating of the sample for the SEM analysis. Traces of K were found in the MgO sample, due to the use of KOH during NP synthesis. The formation of these NPs has also been confirmed from their XRD patterns (Figure 2d,e,f), which demonstrates the hexagonal wurtzite phase of ZnO (ICSD coll. Code 44518) and the cubic phase of both MgO (ICSD coll. Code 9863) and CaO (ICSD coll. Code 28905), respectively.

### 3.2 Characterization of the Biomaterial Inks

#### 3.2.1 FTIR Analysis

**FIGURE 3:**
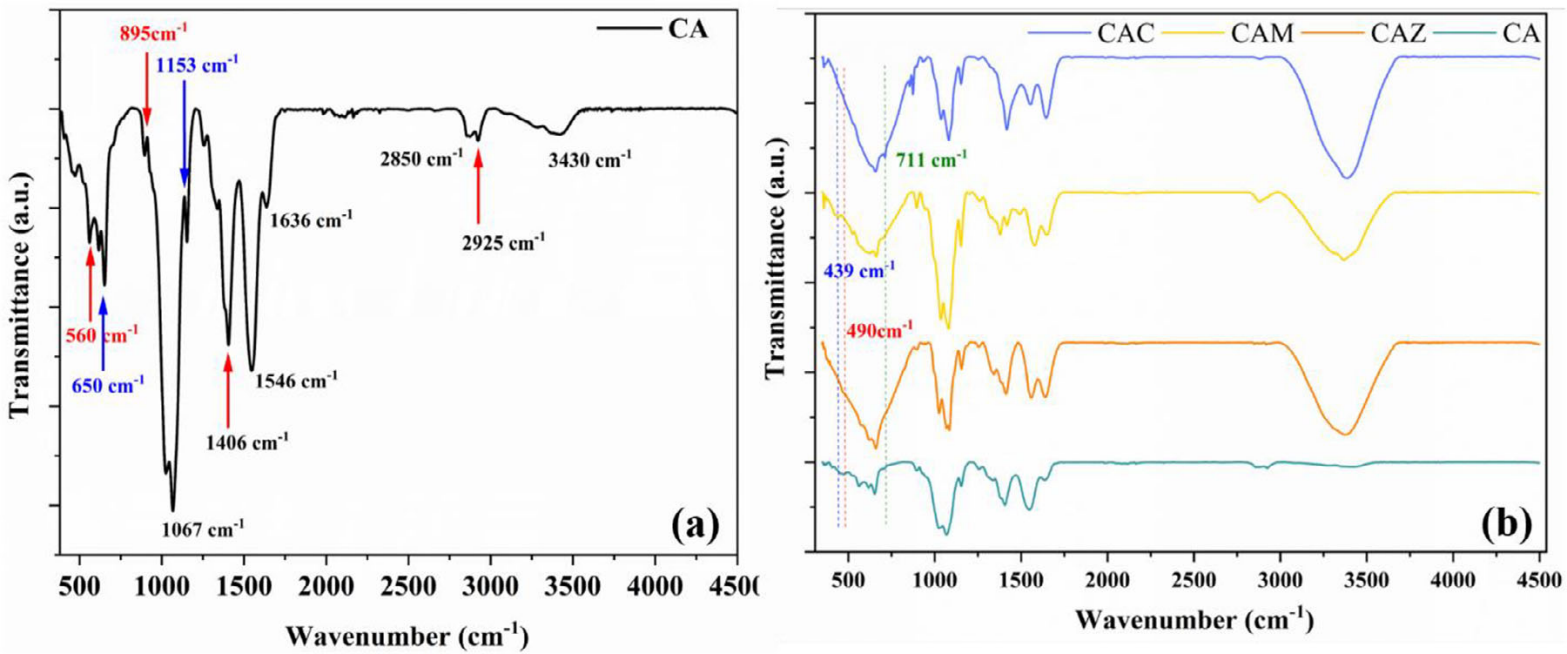
FTIR spectrum of (a) the undoped CA hydrogel and (b) the NPs doped hydrogel samples: CAC (in green), CAM (in blue), CAZ (in red), along with the undoped CA (in black), for comparison.

Figure 3 displays the FTIR spectra of the biomaterial ink hydrogels. For clarity, the FTIR spectrum for the pure CA hydrogel is shown separately in Figure 3a. The spectrum exhibits distinct absorption peaks that align with its molecular composition, encompassing wide bands related to O─H stretching vibrations and the well‐defined peaks that signify the saccharide structure. The blend of CA hydrogel exhibits absorption between 3156 and 3555 cm^−1^, with a peak at 3430 cm^−1^ corresponding to ─NH_2_ and O─H stretching vibrations. The absorptions at 2925 and 2850 cm^−1^ are attributed to the CH_2_ stretching vibrations, whereas the peaks at ∼1636, 1546, and 1406 cm^−1^ are assigned to the amide I, II, and III bands, respectively. The absorption peaks observed at ∼560, 650, and 895 cm^−1^ correspond to the 3,6‐anhydro‐β‐galactose structure of AG in the blend. Additionally, the peaks at 1153 and 1067 cm^−1^ are attributed to the stretching of the C─O bond and the glycosidic linkage present in both CS and AG [22, 23]. With the introduction of NPs (as shown in Figure 3b), the samples containing NPs exhibit a significant enhancement and intensification of peaks in regions related to the hydroxyl and amine functional groups, particularly in CAC. This indicates possible interactions between these groups and the NPs [24, 25], leading to the emergence of fresh bonding contacts inside the hydrogel matrix, potentially impacting its overall structural and mechanical characteristics. The incorporation of CaO nanoparticles into the hydrogel is confirmed by the stretch around 711 cm^−1^ due to Ca─O bonds [26]. The CAM sample, containing MgO NPs, revealed notably sharper peaks in the regions associated with C─O bond stretching and glycosidic linkages. A Mg─O─Mg bond near ∼ 439 cm^−1^ [27] can be seen in the CAM hydrogel. The Zn─O bond near ∼ 490 cm^−1^ [28] in the CAZ sample likely overlapped by polymer bands and is not clearly resolved. The observed improvement in the peak sharpness associated with the C─O bond stretching and the glycosidic linkages, particularly in the CAM samples, indicates greater matrix ordering as a result of the presence of MgO NPs. These interactions are attributed to new bonding and/or alterations in the hydrogen bonding network, resulting in a more compact and interconnected hydrogel network that may impact the mechanical characteristics of the scaffolds [29, 30].

**FIGURE 4:**
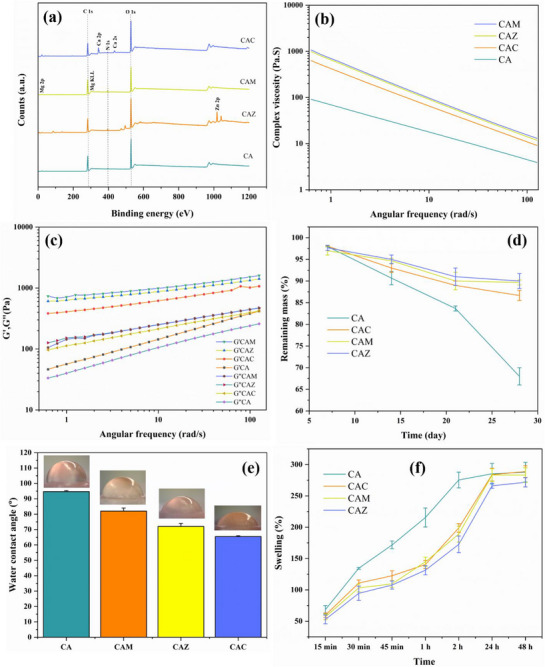
(a) XPS spectra of the samples. (b) Complex viscosity as a function of the angular frequency of the samples. (c) Storage modulus (G’) and loss modulus (G”) as a function of the angular frequency of the samples. (d) Degradation rate of the different 3D printed scaffolds. (e) Wettability measurements of the samples with inset images displaying droplet profiles. (f) Swelling behavior of the samples in PBS (pH 7.4, 25 °C) over a period of 48 h.

The rheological tests (Figure 4b,c) confirmed the spectroscopic findings, showing that the CAM samples had the maximum viscosity and displayed higher storage and loss moduli compared to the other NP‐incorporated samples. The increased viscosity indicates that the presence of MgO NPs enhances the strength of the network inside the hydrogel matrix, resulting in improved resistance to flow and deformation. The impact of NPs on the surface chemistry of the scaffolds was further verified using XPS analysis.

#### 3.2.2 XPS Analysis

The surface elemental composition and chemical states of the CA hydrogel scaffolds, with and without the inclusion of metal oxide NPs, have been analyzed using XPS (Figure 4a). The XPS results support the FTIR findings and provide additional evidence for the changes in the material composition and surface chemistry caused by NP inclusion. The spectra displayed in Figure 4a demonstrate the existence of carbon (C 1s) and oxygen (O 1s) peaks in all samples, indicating the presence of the hydrogel's organic matrix. The spectrum for the CA sample demonstrates a relatively simple pattern, revealing the main components of the hydrogel matrix. The presence of NPs in the CAZ, CAM, and CAC samples is indicated by the emergence of new peaks corresponding to zinc (Zn 2p) in the CAZ sample, magnesium (Mg 2p) in the CAM sample, and calcium (Ca 2p) in the CAC sample. The presence of these peaks confirms the successful integration of the corresponding metal oxide NPs into the surface of the hydrogel scaffolds. The peak due to nitrogen (N 1s) is attributed to the amide bonds of chitosan in the doped and undoped CA hydrogel. This suggests that nitrogen‐containing functional groups on the surface of CS may interact with the metal oxide NPs to some extent in all samples [31, 32].

#### 3.2.3 Printability and Rheological Properties

In a preliminary phase, different ZnO, MgO, and CaO NPs concentrations were tested to maintain both printability and the structural integrity of the printed scaffolds. Tested concentrations of the NPs were 0.3% w/v (low), 0.5% w/v (medium), and 0.7% w/v (high). The results showed that the high concentration was less suitable for printing, leading to either poor structural integrity or printability issues. Additionally, the 0.3% concentration of NPs did not yield significant antibacterial effects (data not shown). Therefore, for the final formulations, we chose the medium concentration of 0.5% w/v (50 mg per 10 mL of hydrogel), as indicated in Table 2. This concentration facilitates an optimal balance between suitable scaffold characteristics and effective printability.

The rheological properties of the CA hydrogel ink with and without NPs additions were evaluated by measuring its complex viscosity at various angular frequencies ranging from 0.628 to 125.704 rad/s. The findings, as illustrated in Figure 4b, indicate that all samples exhibit shear‐thinning characteristics, with the complex viscosity declining as the angular frequency rises. This phenomenon is commonly observed in non‐Newtonian polymeric materials, where the arrangement of polymer chains becomes more pronounced with greater rates of shear [33]. The complex viscosity of the CA sample was the lowest among all the studied materials across the full frequency range. Thus, the CA sample exhibited the most significant shear‐thinning activity. Conversely, the addition of NPs to the CAZ, CAM, and CAC samples led to higher complex viscosities. This indicates that the NPs enhance the overall resistance to flow in these inks. Significantly, the CAM sample exhibited the highest complex viscosity at lower frequencies. The complex viscosity of the CAZ and CAC samples followed similar patterns, with values higher than the pure CA ink but lower than the CAM ink over the entire range of frequencies examined. These results suggest that the NP addition strongly affects the rheological properties, which is anticipated to affect 3D printing processing and the mechanical performance of the printed scaffolds. The observed decrease in viscosity with increasing angular frequency in all formulations (Figure 4b) is in line with prior research on polymer‐NPs composites. This phenomenon is attributed to the influence of NPs on the viscosity and viscoelastic properties of the matrix [34]. The heightened complex viscosity is seen in the inks containing NPs, especially in the CAM sample. Previous studies demonstrate that including NPs into a polymeric network can strengthen the material's ability to withstand deformation. The NPs are responsible for forming physical connections between the polymer chains, resulting in an overall increase in viscosity [35, 36, 37]. Nevertheless, the CAZ and CAC samples exhibited a lower magnitude of alteration in viscosity when compared to CAM, potentially due to the dimensions, configuration, and unique surface contacts of ZnO and CaO NPs within the CS‐AG matrix [38, 39, 40].

The *G*' and *G*'' of the CA hydrogel and its NPs‐modified versions were also measured over a range of angular frequencies from 0.628 to 125.704 rad/s. Figure 4c demonstrates that all samples displayed a characteristic viscoelastic response, with both moduli increasing with the increase in the angular frequency. This indicates that the materials exhibit more elasticity with the rise in the deformation rates. This characteristic is advantageous for bone regeneration scaffolds because it indicates that the materials are capable of withstanding mechanical loads, which is essential for preserving structural integrity when placed in a bone defect site [41]. The CA sample had the lowest *G*' and *G*'' values over the frequency range, suggesting that it is the most pliable and least resilient material among the samples studied. An augmentation in both storage and loss moduli was seen upon the integration of NPs. The CAM sample consistently exhibited the greatest G' values throughout the entire frequency range, inferring that the presence of MgO NPs has a substantial impact on the hydrogel matrix's elasticity and stiffness. Similarly, the CAZ sample and the CAC sample exhibited increased values of *G*' and *G*'' in comparison to those of the pure CA hydrogel. The CAZ sample had a slightly higher modulus than the CAC sample, but both were lower than that of the CAM sample, indicating that ZnO NPs do not significantly change the basic rheological properties of the hydrogel. These findings suggest that although all types of NPs contribute to an augmentation in the viscoelastic characteristics, MgO NPs have a more noticeable impact. G'', which characterizes the viscous properties of the materials, exhibited a comparable pattern to the storage modulus, with the CAM sample displaying the largest magnitudes. A greater G' value for CAM suggests that MgO NPs play a more substantial role in the scaffold's elasticity, possibly due to more robust network development in the hydrogel [35, 36]. This finding demonstrates that although the materials have characteristics of a solid at higher frequencies, they still possess an inherent viscosity that contributes to the overall viscoelastic behavior.

The difference in the moduli indicates that the scaffolds have characteristics of both solids and liquids, which can be beneficial for the 3D printing process by enabling easy extrusion and positioning of the hydrogel ink. The graph shows that NPs have a stabilizing influence on the viscoelastic characteristics. No crossover between *G*' and *G*″ was detected, indicating that the scaffolds predominantly maintain their elastic properties across the investigated frequency range. The stabilization may result from the contact between the NPs and the hydrogel matrix, potentially including physical entanglements or ionic interactions that strengthen the network's structure [42]. No crossover between G′ and G″ was detected, indicating that the scaffolds predominantly maintain elastic behavior across the investigated frequency range. The rheological parameters observed here align with the chosen printing parameters for each formulation. Although NP‐induced viscosity increases could impede printing, the adjusted extruder temperature and pressure = = mitigated these effects, enabling smooth extrusion and good shape fidelity, as demonstrated by the parameters in Table 3. The ability to shear‐thin and rapidly recover is crucial for high‐resolution scaffold fabrication [41]. The variations in viscoelastic behavior across formulations support selecting specific NPs to tune scaffold mechanics for tissue‐engineering needs [43].

#### 3.2.4 Hydrolytic Degradability and Cross‐Linking Density Measurement

Figure 4d depicts the in vitro degradation pattern of different scaffolds in PBS for a period of 28 days. The remaining mass (%) is utilized to compare the degradation rates of different scaffold formulations. CA exhibited the fastest degradation rate compared to the other samples, showing a consistent decrease in mass throughout the study period, as anticipated for this naturally produced material undergoing hydrolytic degradation in PBS [44]. The results showed that incorporating NPs into the CA hydrogel significantly slowed down the breakdown rate. CAC was found to slow down the breakdown rate of the hydrogel matrix compared to CA. This may be caused by the ionic crosslinking effect of calcium ions, which can create connections between the polymeric chains, strengthening the hydrogel's structure and making it less prone to breakdown [45]. Consistent with existing literature, calcium ions have been demonstrated to engage with biopolymer matrix, thereby decelerating the hydrolysis process [46, 47, 48]. The decreased disintegration rate in the CAM scaffolds indicates that MgO may provide extra physical stability to the scaffold [49]. Magnesium ions, similar to calcium, may engage in ionic crosslinking or interact with water molecules to enhance the stability of the hydrogel network against degradation [50]. CAZ scaffolds also showed a substantial decrease in the degradation rate compared to CA. Previous studies confirmed that zinc may stabilize the hydrogel matrix by potentially creating complexes with the polymer chains, which restricts the entry of the degrading substances. The gradual breakdown of the CAZ scaffolds may be advantageous in situations where extended implant durability is needed until full tissue regeneration takes place [51, 52, 53, 54].

The structural changes during degradation were further visualized through SEM imaging of scaffolds at different time points (weeks 1 to 4) and the results are presented in Table 5. For the CA group, the surface morphology showed noticeable structural deterioration over time. By week 4, the scaffolds exhibited an eroded surface with a loose and porous architecture, consistent with the rapid degradation trend observed in the degradation study. The increase in porosity and disruption of the surface indicate significant matrix breakdown, likely due to the hydrolytic degradation of the biopolymer backbone. In contrast, CAC scaffolds maintained a more compact and intact microstructure across the same period. While minor surface erosion was observed, the scaffold integrity was largely preserved even at week 4. This observation supports the role of Ca^2^⁺ ions in enhancing the physical stability of the hydrogel via ionic crosslinking [55], as previously discussed. CAM scaffolds also retained their structural integrity better than the CA group. Although some porosity appeared over time, the SEM images showed less collapse or fragmentation. The presence of Mg^2^⁺ may have contributed to a denser internal network [50], providing resistance against degradation‐induced mechanical weakening. For CAZ scaffolds, SEM images revealed moderate degradation features. The surface became increasingly porous over the 4‐week period, but the extent of disintegration remained lower than that of the CA group. This behavior can be attributed to Zn^2^⁺ interactions with polymer chains, which possibly restrict the accessibility of degrading agents [56]. Overall, SEM imaging supports the quantitative degradation findings by visually confirming that scaffolds with incorporated oxide nanoparticles (CaO, MgO, ZnO) demonstrate improved resistance to structural deterioration compared to pure CA. These results highlight the importance of NP‐mediated stabilization for achieving long‐term scaffold integrity in physiological conditions.

**TABLE 5:** Representative SEM images of 3D‐printed scaffolds (CA, CAC, CAM, and CAZ) after 1, 2, 3, and 4 weeks of incubation in PBS. Scale bar: 500 µm.

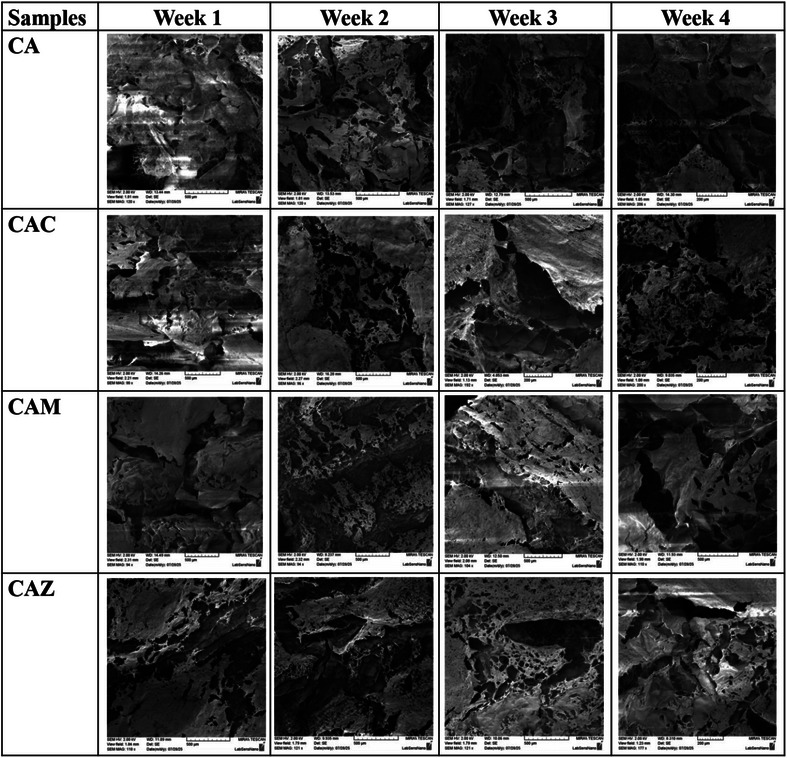

Cross‐linking density results further support these findings. The calculated Ve values (Table 6) revealed that CAZ exhibited the highest cross‐linking density (0.0443 mol/cm^3^), while CA showed the lowest (0.0431 mol/cm^3^). CAC and CAM presented intermediate values of 0.0430 and 0.0434 mol/cm^3^, respectively. This trend aligns with the observed decrease in swelling and degradation rates upon NP incorporation, indicating that Zn^2^⁺ ions may form more stable coordination bonds with the polymer network [57], whereas Mg^2^⁺ and Ca^2^⁺ contribute to physical ionic interactions that enhance structural integrity [58]. Although CAM showed the highest viscosity in rheological analysis, its Ve value was not the highest, suggesting that MgO primarily influences short‐term viscoelasticity through reversible ionic bridging, whereas ZnO provides more permanent interactions that restrict water uptake. Overall, these data confirm that NP‐induced interactions increase the effective cross‐linking density, thereby improving scaffold stability while maintaining sufficient hydration for cellular activity.

**TABLE 6:** Average dry weight (Wi), swollen weight (Ww), swelling ratio (Q), polymer volume fraction (Vr), and calculated cross‐linking density (Ve) of CA, CAZ, CAC, and CAM scaffolds determined using the Flory–Rehner equation. Values are based on equilibrium swelling measurements in PBS (pH 7.4) at 25°C.

Sample	Wi (g)	Ww (g)	Q (Ws/Wd)	Vr	Ve (mol/cm^3^)
CA	0.11587	0.44900	3.87514	0.20512	0.04313
CAC	0.11527	0.44800	3.88664	0.20464	0.04304
CAM	0.11557	0.44267	3.83040	0.20702	0.04344
CAZ	0.11547	0.42900	3.71536	0.21207	0.04426

#### 3.2.5 Wettability Measurement

The surface wettability of the hydrogel inks was assessed using water contact angle measurements. As shown in Figure 4e, there is a clear trend toward greater hydrophilicity upon NP incorporation. The contact angle of CA was 94.7°, suggesting a slight hydrophobic characteristic. Introducing NPs resulted in a significant reduction in contact angle values. The CAM sample, containing MgO NPs, exhibited a contact angle of 82°, while the CAZ sample, containing ZnO NPs, had a contact angle of 72°. The CAC sample, which consists of CaO NPs, displayed the lowest contact angle of 65.5°. The data validate the notion that the addition of metal oxide NPs to the hydrogel matrix increases its hydrophilicity [59].

The degree of water attraction on the surface of scaffolds is vital for the cellular processes that are important for tissue integration and regeneration. The addition of CaO NPs to the CA scaffolds resulted in significant improvements in hydrophilicity. This can be attributed to the hygroscopic properties of calcium ions, which form multiple hydrogen bonds with water molecules. As a result, the surface of the scaffolds becomes more suitable for cell attachment and growth, resembling the natural extracellular matrix [60, 61, 62]. The incorporation of ZnO NPs in the CA scaffolds enhanced hydrophilicity by forming polar interactions with water, thereby exhibiting the potential to improve cell adhesion, transport nutrients, and activate genes associated with cell proliferation [63, 64, 65]. The CAM scaffolds displayed more hydrophilicity than the unmodified CA hydrogel, but to a smaller degree in comparison to that of CAZ and CAC scaffolds. The concentration of MgO NPs used may not have enhanced water contact as efficiently as CaO and ZnO, either due to their surface chemistry restricting hydrogen bonding sites or resulting in less orderly configurations of water molecules at the interface [66]. Additionally, NP size may contribute; smaller NPs offer greater surface area for water interaction and thus higher apparent hydrophilicity [67]. This emphasizes the interplay between scaffold chemistry/physics and water at the interface.

#### 3.2.6 Swelling Behavior

Figure 4f illustrates the swelling profiles of the CA, CAZ, CAC, and CAM scaffolds in PBS over 48 h. All samples exhibited a rapid initial uptake of water within the first 2 h, followed by a gradual increase until equilibrium was reached at 24–48 h. The control CA scaffold demonstrated the highest swelling percentage (∼300%), reflecting its less compact structure and absence of reinforcing agents. In contrast, scaffolds containing oxide NPs showed significantly lower swelling ratios compared to CA, suggesting that the incorporation of NPs enhanced the network density and reduced free volume available for water penetration. Among the NP‐reinforced scaffolds, CAM exhibited the lowest swelling capacity, which may be attributed to the strong ionic interactions and hydrogen bonding of Mg^2^⁺ ions with the polymeric chains, leading to a more physically crosslinked structure. CAC also showed reduced swelling compared to CA, likely due to additional ionic crosslinking from calcium ions that stabilized the CS–AG network [45]. Similarly, CAZ displayed restricted swelling, as Zn^2^⁺ ions may form coordination complexes with polymer chains, thereby reducing chain mobility. These observations align with the degradation results, where the incorporation of NPs significantly slowed matrix breakdown, reinforcing the hypothesis that NPs strengthen the hydrogel network through secondary interactions [68]. Reduced swelling in these scaffolds can be advantageous for maintaining dimensional stability and mechanical integrity during implantation, while still allowing sufficient water uptake for nutrient diffusion and cell viability.

### 3.3 Cell Proliferation

**FIGURE 5:**
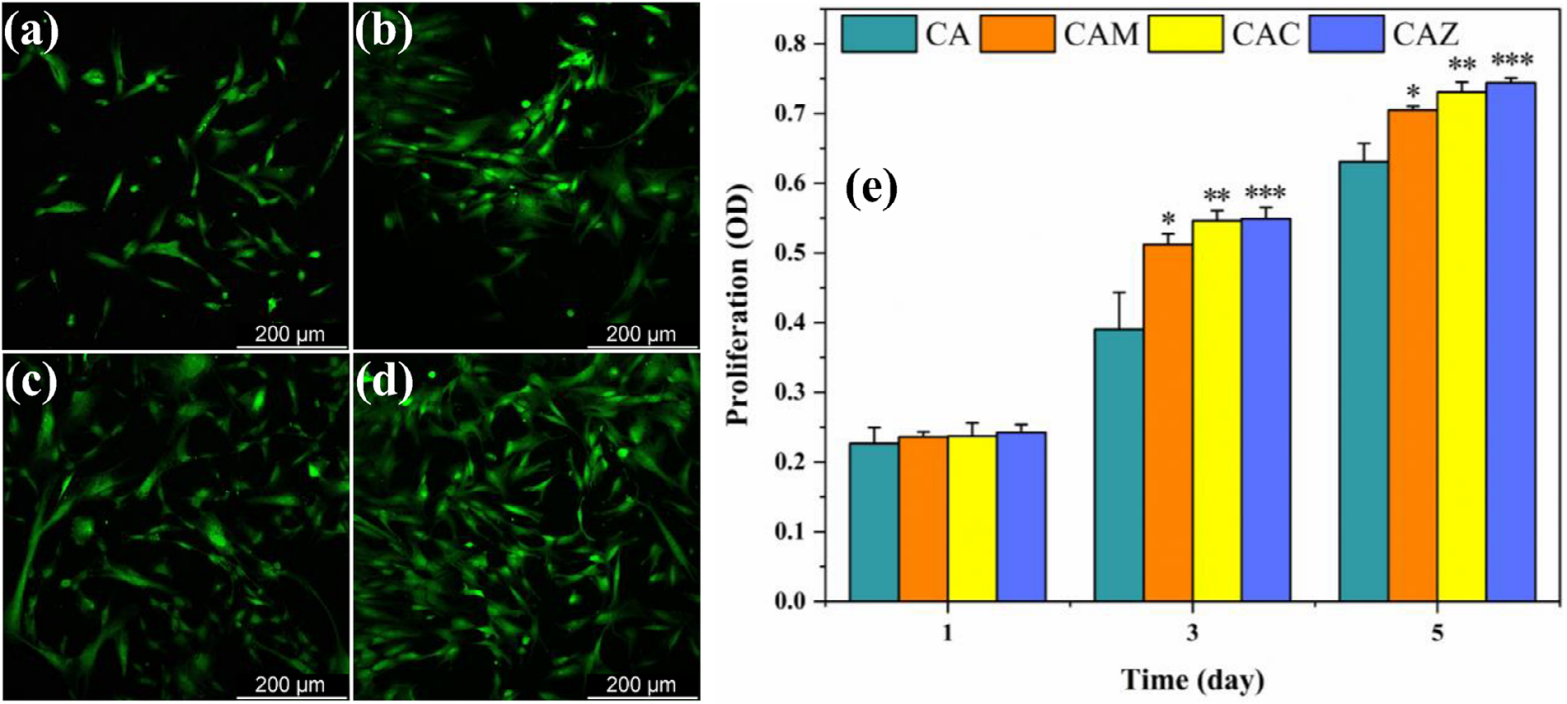
Confocal microscope image of cultured BMSCs on the surface of (a) CA, (b) CAM, (c) CAC, and (d) CAZ scaffolds on day 2. (e) The results of the quantitative MTT experiment demonstrate cell proliferation over 5 days. Data are presented as mean ± SD; n = 3. ^*^ *p *<0.05, ^**^ *p *<0.01, and ^***^ *p *<0.001 compared to CA.

Figure 5 displays the growth evaluation of BMSCs on the scaffolds. Confocal fluorescence microscopy images (Figure 5a–d) exhibit the morphological traits of BMSCs at a magnification that allows the visualization of cell spreading and distribution on the scaffolds. The MTT assay results provided a quantifiable measurement of cell proliferation over a 5‐day timeframe (Figure 5e). The data revealed consistently lower optical density (OD) readings for the CA scaffold compared to the NP‐doped CA scaffolds, indicating slower cell proliferation over the five days. Conversely, the NP‐doped scaffolds demonstrate improved cell growth. CAZ and CAC showed greater cell density and enhanced spreading (Figure 5e,c,d) compared to CAM (Figure 5e,b), which exhibited a modest decline. All NP‐doped scaffolds considerably surpass the control CA scaffold in promoting cell proliferation. The observed differences in cell proliferation could be influenced by the extent of ion release, stimulating the cell metabolism. Calcium ions regulate multiple cellular activities, such as adhesion, proliferation, and differentiation [69, 70, 71]. Zinc ions act as a structural signal for cellular attachment and may also activate genes that control cell proliferation and differentiation [72]. Magnesium ions are important for maintaining the cell cycle, proliferation, and differentiation [73]. Thus, all metal ions acted with their different strength and at the various levels of cell metabolism with positive efficacy. While the scaffolds supported BMSC attachment and proliferation on their surface, we observed limited evidence of deep cell infiltration. This may be due to the dense hydrogel network, which can restrict cellular migration into the inner layers. Future work may employ scaffold design changes or imaging to promote and assess deeper infiltration.

### 3.4 Osteogenic Differentiation

**FIGURE 6:**
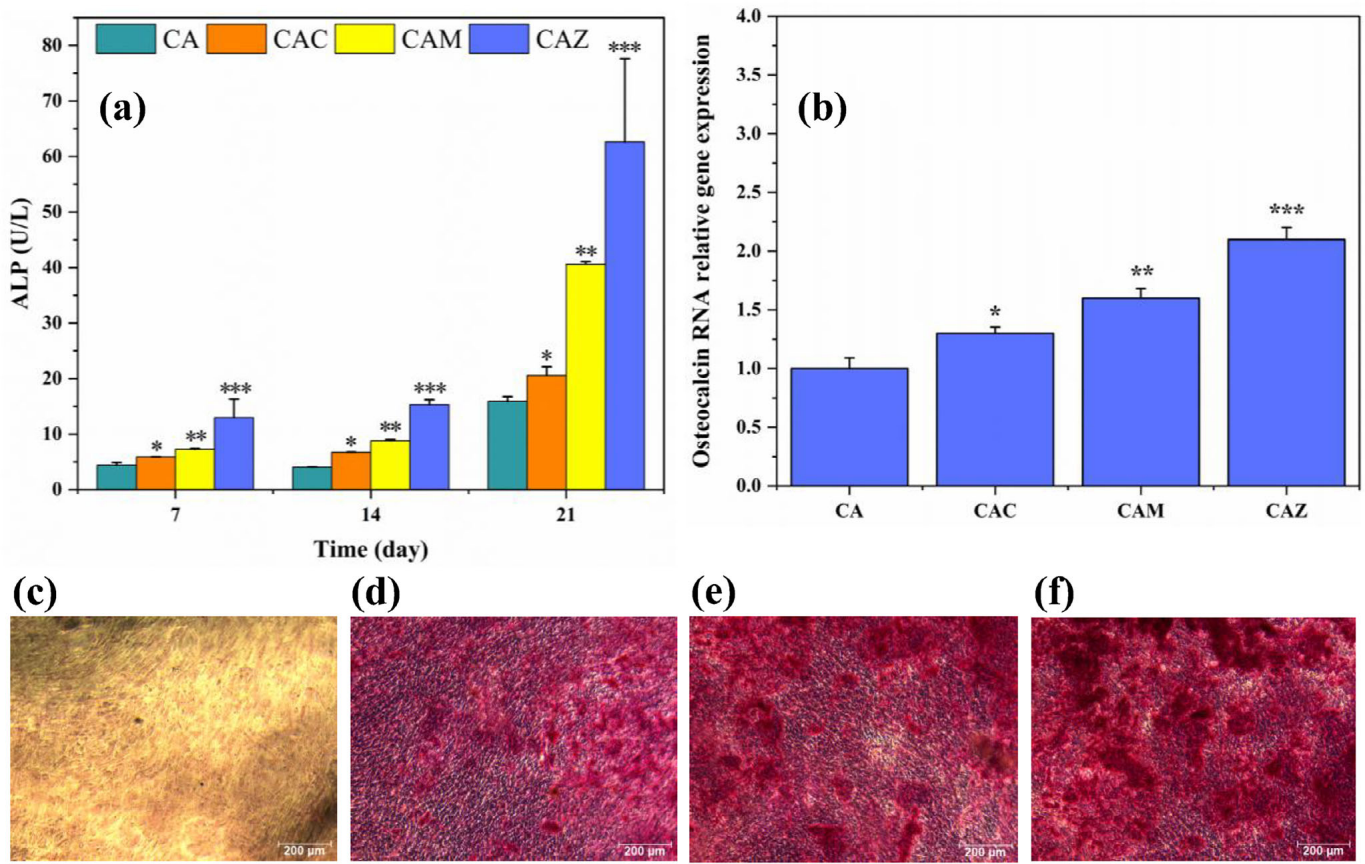
(a) ALP activity of the samples at 7, 14, and 21 days after incubation. Data are presented as mean ± SD; n = 3. ^*^ *p *< 0.05 compared to CA^0^ *p *< 0.05, ^**^ *p *< 0.01, and ^***^ *p *< 0.001 compared to CA. (b) The qRT‐PCR results showing the relative gene expression of the osteocalcin gene (307 bp) normalized to the GAPDH gene (319 bp) as an indicator of osteogenic differentiation across scaffold types CA, CAC, CAM, and CAZ. Data are presented as mean ± SD; *n* = 3. ^*^ *p *< 0.05, ^**^ *p *< 0.01, and ^***^ *p *< 0.001 compared to CA. (c–f) Representative phase contrast microscope images of ARS staining of CA (c), CAM (d), CAZ (e), and CAC (f) after 21 days of culture, illustrate the deposition of minerals on scaffolds. The variable intensities of red staining indicate different levels of calcium buildup. Scale bars: 200 µm.

The role of metal ions in osteogenic differentiation and bone formation is highly complex and multifactorial. A wide spectrum of inorganic ions, including calcium, magnesium, zinc, phosphate, and titanium is involved [74]. Figure 6 illustrates osteogenic differentiation by correlating ALP activity, osteocalcin gene expression, and mineralization of scaffolds that release calcium, magnesium, and zinc ions. Figure 6a shows ALP activity measurements from all samples over 21 days. ALP activity is critical for osteogenic differentiation and bone mineralization. Cells exhibited negligible ALP activity on days 7 and 14 across all scaffolds, indicating similar early cellular activity. However, by day 21 ALP activity increased substantially; for CAZ and CAM it rose approximately 4–5‐fold relative to day 7. CAZ scaffolds had the highest mean ALP activity, but the large standard deviation adds variability, so the difference from CAM scaffold less pronounced. Figure 6b depicts a qRT‐PCR assessment of osteocalcin expression, a suitable marker for bone formation. The osteocalcin relative expression, normalized to GAPDH, indicates differential effects on osteogenic differentiation among scaffold types. CAZ had the highest expression followed by CAC and CAM, respectively, which is consistent with the literature. Zinc ions have been found to enhance bone formation by acting as a cofactor for enzymes like ALP as well as stimulating transcription factors critical for osteoblast gene expression [75, 76]. Magnesium ions also act as cofactors for ALP enzymes [77], and can enhance osteocalcin expression, although typically with a smaller effect than zinc. The calcium ions in CAC have less direct impact on the osteoblast activity but are more strongly influenced on the mineral deposition. Figure 6c–f represents the ARS staining assay for mineralization via calcium deposition as indicators of osteoblast precursors and mineralization. Figure 6c shows ARS staining limited to the CA scaffold, which suggests less calcium deposition. Meanwhile, in Figure 6d,e, CAM and CAZ, respectively, show moderate staining as indicated by red coloration, implying moderate calcium deposition. Figure 6f demonstrates CAC with the highest ARS staining intensity, suggesting the most extensive calcium deposition among the samples. The outcomes correlate with ALP activity and osteocalcin expression levels, indicating that the NP‐doped scaffolds contributed to greater mineralization, and the introduction of NPs significantly enhanced osteogenic differentiation. Zinc and magnesium ions primarily affected early osteogenic markers (ALP, osteocalcin), whereas calcium ions mainly supported the mineral composition, while still contributing to overall scaffold performance. The results show the importance of selecting suitable NPs to achieve increased biological efficacy with improved scaffold performance in bone tissue engineering.

### 3.5 Antibacterial Analysis

**FIGURE 7:**
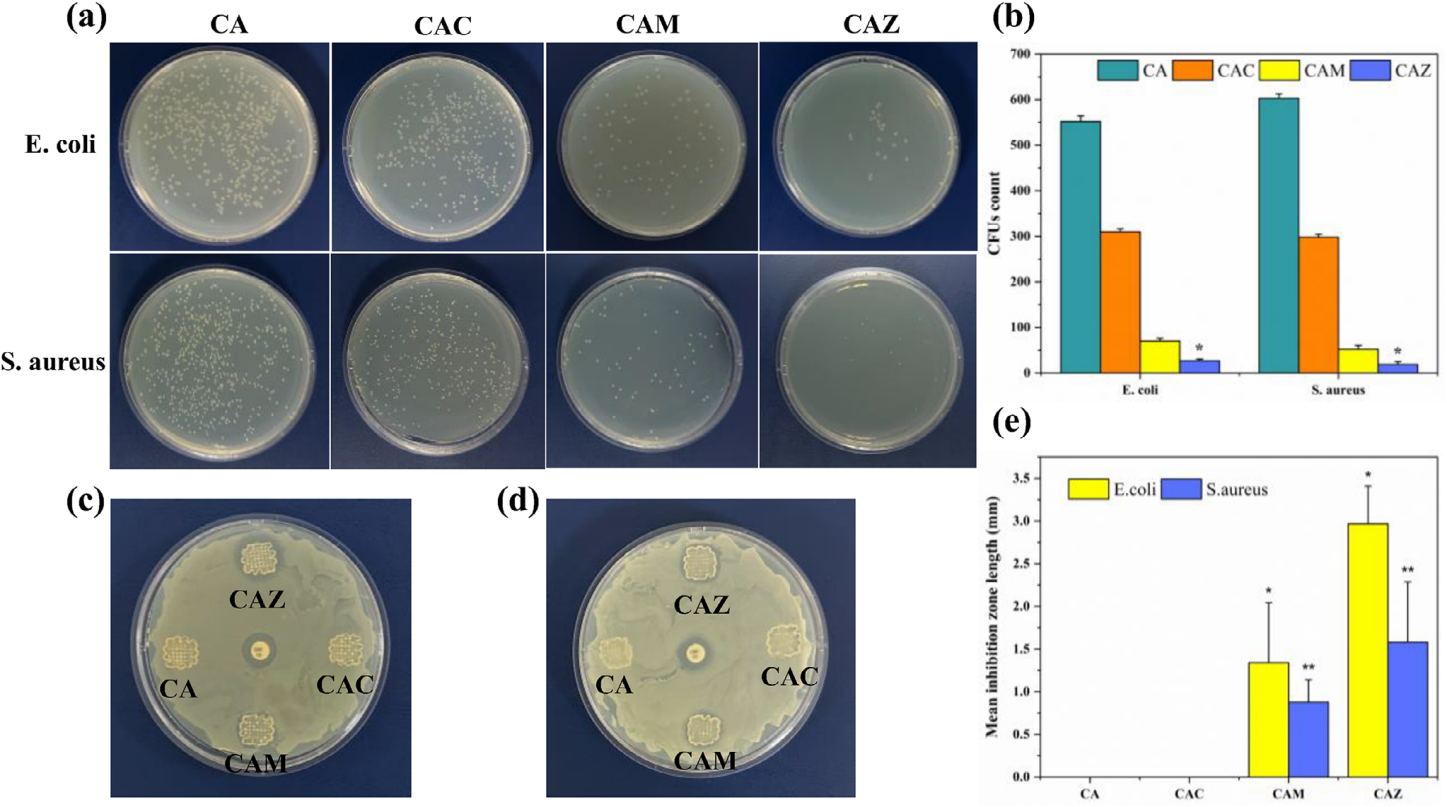
(a) Comparative antibacterial efficacy of CA hydrogel vs CA hydrogel infused with ZnO (CAZ), CaO (CAC), and MgO (CAM) NPs against *E. coli* and *S. aureus*. The top and bottom rows display CFUs for each hydrogel formulation after 24 h of incubation. (b) The bar graph counts the number of CFUs, using the CA hydrogel ink as a control. (c,d) Disc diffusion method for *E. coli* and *S. aureus*. (e) Quantitative evaluation of the disc method. Data are presented as mean ± SD; n = 3. ^*^ *p *< 0.05 compared to CA, ^**^ *p *< 0.01 compared to CA.

The effectiveness of the scaffolds against *E. coli* and *S. aureus* was tested to determine their antibacterial properties. Figure 7a,b displays the results of the plate count method, showing the bacterial colonies after 24 h of incubation. The CA scaffold showed a notable number of colonies for both bacterial strains, suggesting low intrinsic antibacterial properties. CAZ and CAM exhibited a significant decrease in *E. coli* colony numbers (approximately 11‐fold drop in value) compared to CA, indicating improved antibacterial characteristics. CAC showed an approximately 50% reduction in bacterial colonies compared to CA. Similar results were obtained for Gram‐positive bacteria. The study indicates that adding ZnO and MgO NPs to the CA hydrogel matrix greatly improves the antibacterial characteristics of the ink, with ZnO NPs having the strongest impact against *S. aureus* and *E. coli*. Further examination of the scaffolds’ antibacterial activity was evaluated by the disc diffusion method. The results clearly indicate the better antibacterial properties of the CAZ scaffold, which showed considerable suppression against *E. coli* (Figure 7c) and *S. aureus* (Figure 7d). CAM also exhibited antibacterial properties, albeit to a lesser degree than CAZ. CAC and CA scaffolds showed no inhibition, highlighting the limited antibacterial efficacy of CaO NPs and the natural hydrogel matrix without NPs augmentation. The inhibition zones were quantified by measuring the distance from the edge of the sample to the edge of the inhibition zone at twelve points around each sample using ImageJ software. The average inhibition lengths for *S. aureus* were 1.58 mm for CAZ and 0.87 mm for CAM, while for *E. coli*, the values were 2.96 mm for CAZ and 1.34 mm for CAM. The inhibition zones for the gentamicin antibiotic discs (positive control) were measured to be 3.47 mm for *E. coli* and 3.43 mm for *S. aureus*. Although the antibacterial effect of inorganic NPs and their proposed mechanism have been widely reported, the antibacterial efficacy can vary by specific samples and must always be experimentally tested. For instance, zinc oxide NPs are well‐known for their strong antibacterial properties against both Gram‐positive and Gram‐negative bacteria, mainly due to the release of zinc ions (Zn^2+^) and the formation of reactive oxygen species (ROS) when they come into contact with bacteria [78]. Zinc ions disrupt the integrity of bacterial cell membranes, while ROS cause oxidative stress, thereby causing damage to the bacterial DNA, proteins, and lipids, ultimately inhibiting bacterial growth [79]. In our work, CAM scaffolds have demonstrated antibacterial effectiveness, outperforming CAC scaffolds. This increased activity may be associated with the liberation of magnesium ions (Mg^2+^), which could interfere with the bacterial cell membrane potential, resulting in cell demise [80]. CAC also exhibits enhanced antibacterial properties when compared to CA, mainly by releasing calcium ions (Ca^2+^). These ions have the ability to interfere with the function and composition of bacterial cell walls, particularly in Gram‐positive bacteria such as *S. aureus*. However, at the concentrations used here, the antibacterial effect of Ca^2^⁺ appears weaker than the combined actions of ZnO and MgO NPs [81].

## 4 Future Directions and Improvements

To further advance this research, several key steps should be implemented. First, in vivo studies are essential for assessing the efficacy of these NP‐doped scaffolds in bone healing contexts. Given the encouraging in vitro findings, especially concerning the CAZ scaffold, future research could investigate its in vivo performance utilizing a critical‐sized bone defect model in rats. This approach would provide insights into bone regeneration, scaffold integration, and any immune responses triggered by the embedded NPs. Second, optimizing the scaffold composition could lead to enhancements in both mechanical strength and bioactivity. For instance, a slightly higher polymer concentration or a mild chemical crosslink (e.g., using genipin) could be introduced to increase stiffness for load‐bearing needs while preserving pore size for infiltration. Additionally, tuning the NPs content (or combining multiple types in one scaffold) is an interesting route; our study kept NP content constant at 0.5%, but perhaps a gradient or a layered scaffold with different NPs in different regions could synergistically provide antimicrobial action (ZnO, MgO) and osteoinduction (Ca^2+^ from CaO) in a spatially targeted manner. Third, incorporation of growth factors or biologics can be considered: the free amine groups on chitosan (≈80% free as measured) offer anchoring points for tethering molecules like BMP‐2 or VEGF [82]. This could enhance the osteogenic or angiogenic capacity of the scaffold beyond what the inorganic components alone achieve. Lastly, advanced scaffold designs such as core–shell fibers (with different materials in the core vs shell) or printed channels to promote vascularization could be explored to further improve cell invasion and nutrient delivery. These future improvements, combined with the current finding that a 3D‐printed CS‐AG scaffold can be successfully doped with bioactive NPs, will guide the development of next generation hybrid biomaterials for bone tissue engineering.

## 5 Conclusion

This study provides a comparative analysis of ZnO, MgO, and CaO NPs in the 3D‐printed CS‐AG scaffolds. A comprehensive evaluation of the physicochemical and biological properties of the NPs and scaffolds has been performed and discussed. The optimized composition of the biomaterial ink for printing was 1.5 % AG, 3.5 % CS and 0.5% of NPs. The incorporation of NPs into the CA hydrogel reduced degradability and increased complex viscosity. ZnO NPs were particularly effective in improving antibacterial characteristics and promoting possible bone regeneration when incorporated into the scaffolds. ZnO NPs outperformed MgO and CaO NPs in antibacterial efficacy against selected Gram‐positive and Gram‐negative pathogens, as well as in enhancing the proliferation and osteogenic differentiation of bone marrow stem cells, as demonstrated by ALP activity and qRT‐PCR. The findings indicate that ZnO‐doped scaffolds offer a promising solution to both infection control and bone healing. Furthermore, the study highlights the significance of appropriate NP selection in the construction of the scaffolds, particularly emphasizing the potential of ZnO NPs for future research in tissue engineering and regenerative medicine.

## Conflicts of Interest

The authors declare no conflicts of interest.

## Data Availability

The data that support the findings of this study are available on request from the corresponding author. The data are not publicly available due to privacy or ethical restrictions.
